# A genetic-algorithm-based remnant grey prediction model for energy demand forecasting

**DOI:** 10.1371/journal.pone.0185478

**Published:** 2017-10-05

**Authors:** Yi-Chung Hu

**Affiliations:** 1 College of Management & College of Tourism, Fujian Agriculture and Forestry University, Fuzhou City, China; 2 Department of Business Administration, Chung Yuan Christian University, Taoyuan City, Taiwan; Chongqing University, CHINA

## Abstract

Energy demand is an important economic index, and demand forecasting has played a significant role in drawing up energy development plans for cities or countries. As the use of large datasets and statistical assumptions is often impractical to forecast energy demand, the GM(1,1) model is commonly used because of its simplicity and ability to characterize an unknown system by using a limited number of data points to construct a time series model. This paper proposes a genetic-algorithm-based remnant GM(1,1) (GARGM(1,1)) with sign estimation to further improve the forecasting accuracy of the original GM(1,1) model. The distinctive feature of GARGM(1,1) is that it simultaneously optimizes the parameter specifications of the original and its residual models by using the GA. The results of experiments pertaining to a real case of energy demand in China showed that the proposed GARGM(1,1) outperforms other remnant GM(1,1) variants.

## Introduction

Energy is necessary for the sustainable development and economic prosperity of a country [1], and this is evidenced by the fact that energy demand has emerged as an important economic index in recent years. With the rapid pace of industrialization, the global demand for energy has increased exponentially in the past decade. Worldwide energy consumption is expected to increase by over 50% before 2030 if the current pattern of global energy consumption continues [2]. Moreover, high energy consumption has a significant and deleterious impact on the environment. This means that the environmental impact of energy consumption will play a crucial role in guiding energy development policies for cities and countries in the future [1]. An important issue in this context is the ability to accurately predict energy demand.

Traditional methods of demand forecasting, including artificial intelligence techniques, multivariate regression, and time series analysis, have been frequently applied to predict energy demand [3–8]. However, a large sample size is usually required to achieve reasonable forecasting accuracy for these methods [9–12]. Furthermore, statistical methods usually require that the data conform to statistical assumptions such as following a particular distribution. However, using large sample sizes or conforming to statistical assumptions is often impractical [13]. Hence, a forecasting method is needed that can work with small samples without making statistical assumptions to construct an energy demand prediction model [10, 11].

Grey prediction [14] has emerged as a popular technique in the past decade, and is suitable for forecasting energy demand because of its simplicity and ability to characterize an unknown system using a limited number of data points [1]. Grey prediction consists of several forecasting models, of which the GM(1, 1) is commonly used for time series forecasting [15]. The GM(1, 1) model needs only four recent sample data points to achieve reliable and acceptable prediction accuracy [16, 17]. Its effectiveness has been verified through application to a wide range of real-world problems, including energy consumption forecasting [10–13, 18–23], technology management [24, 25], engineering problems [26], optimization model development [27, 28], and general management [29–31].

To increase the forecasting accuracy of the original GM(1, 1) model, further development of the residual GM(1,1) model has been recommended [14, 15]. A residual modification model, also called a remnant GM(1,1) model, is commonly constructed by first building the original GM(1,1) model, and then constructing the residual GM(1,1) model to modify the predicted values obtained by the original model. A number of improved remnant GM(1, 1) models focusing on sign estimation for residual modification have been developed. For instance, Hsu and Chen [32] used a multi-layer perceptron (MLP) to estimate the signs of residual modification to forecast power demand; Hsu [33] used Markov-chain-based sign estimation to modify residuals for the global integrated circuit industry, whereas Lee and Tong [13] combined residual modification with residual genetic programming (GP) sign estimation to develop the GPGM(1, 1) model in view of the importance of forecasting energy demand.

Usually, the original and the residual models are set up separately for remnant models. It would be interesting to investigate whether the prediction accuracy of the traditional remnant GM(1,1) model improves when the GM(1,1) and its residual models are constructed simultaneously. This paper develops a grey forecasting model called the genetic-algorithm-based remnant GM(1,1) model (GARGM(1,1)) with sign estimation that delivers high prediction accuracy. Its distinctive feature is that it can simultaneously optimize the parameters required for the original GM(1,1) and its residual models by a powerful search and optimization method [34–36], the genetic algorithm (GA). This grey prediction model is then applied to forecast energy demand.

The remainder of the paper is organized as follows. Sections 2 and 3 introduce the traditional remnant GM(1,1) and the proposed GARGM(1,1) models, respectively. Section 4 examines the forecasting performance of the GARGM(1,1) model using a dataset collected from China Statistical Yearbook 2008. The results show that the GARGM(1,1) model can outperform other variants of the remnant GM(1,1) model. Section 5 contains a discussion and the conclusions of this study.

## Remnant GM(1,1) model

This section introduces the traditional remnant GM(1,1) model used to improve the predictive accuracy of the original GM(1,1) model. It consists of two main components: the original GM(1,1) model described in Section 2.1, and the residual GM(1,1) model described in Section 2.2.

### Original GM(1,1) model

Let an original data sequence $x_{}^{\left(0\right)}=(x_{1}^{\left(0\right)},x_{2}^{\left(0\right)},\ldots ,x_{n}^{\left(0\right)})$ be provided by one system and consist of *n* samples. A new sequence $x_{}^{\left(1\right)}=(x_{1}^{\left(1\right)},x_{2}^{\left(1\right)},\ldots ,x_{n}^{\left(1\right)})$ can then be generated from **x**^(0)^ by the accumulated generating operation (AGO) [7, 15] as follows:

$$x_{k}^{\left(1\right)}=\sum_{j=1}^{k}x_{k}^{\left(0\right)},k=1,\ldots ,n$$ (1)

$x_{1}^{\left(1\right)}$, $x_{2}^{\left(1\right)}$,…, $x_{n}^{\left(1\right)}$ can then be approximated by a first-order differential equation:

$$\frac{dx^{\left(1\right)}}{dt}+ax^{\left(1\right)}=b$$ (2)

where *a* and *b* are the developing coefficient and the control variable, respectively. The AGO is used because it can identify potential regularities hidden in the data sequences even if the original data are finite, insufficient, and chaotic.

${\hat{x}}_{k}^{\left(1\right)}$, the predicted value of $x_{k}^{\left(1\right)}$, can be obtained by solving the grey difference equation with initial condition $x_{1}^{\left(1\right)}=x_{1}^{\left(0\right)}$:

$${\hat{x}}_{k}^{\left(1\right)}=(x_{1}^{\left(0\right)}-\frac{b}{a})e^{-a(k-1)}+\frac{b}{a}$$ (3)

*a* and *b* can be estimated by means of a grey difference equation:

$$x_{k}^{\left(0\right)}+az_{k}^{\left(1\right)}=b$$ (4)

where the background value $z_{k}^{\left(1\right)}$ is formulated as follows:

$$z_{k}^{\left(1\right)}=\alpha x_{k}^{\left(1\right)}+(1-\alpha )x_{k-1}^{\left(1\right)}$$ (5)

*α* is usually specified as 0.5 for convenience, but this is not the optimal setting. By using *n*–1 grey difference equations (*k* = 2, 3,…, *n*), *a* and *b* can be obtained by the ordinary least-squares method:

$${\left[a,b\right]}^{T}={\left(B^{T}B\right)}^{-1}B^{T}y$$ (6)

where

$$B=\left[\begin{array}{cc} -z_{2}^{\left(1\right)} & 1\\ -z_{3}^{\left(1\right)} & 1\\ \vdots & \vdots \\ -z_{n}^{\left(1\right)} & 1 \end{array}\right]$$ (7)

$$y={\left[x_{2}^{\left(0\right)},x_{3}^{\left(0\right)},\ldots ,x_{n}^{\left(0\right)}\right]}^{T}$$ (8)

Using the inverse AGO, the predicted value, ${\hat{x}}_{k}^{\left(0\right)}$, of $x_{k}^{\left(0\right)}$ can be generated as follows:

$${\hat{x}}_{k}^{\left(0\right)}={\hat{x}}_{k}^{\left(1\right)}-{\hat{x}}_{k}^{\left(1\right)},\;k=2,3,\ldots ,n$$ (9)

Therefore, ${\hat{x}}_{k}^{\left(0\right)}$ can be formulated as follows:

$${\hat{x}}_{k}^{\left(0\right)}=(1-e^{a})(x_{1}^{\left(0\right)}-\frac{b}{a})e^{-a(k-1)},k=2,3,\ldots ,n$$ (10)

Note that ${\hat{x}}_{1}^{\left(1\right)}={\hat{x}}_{1}^{\left(0\right)}$.

### Residual GM(1,1) model

Let **ε**^(0)^ = ($\varepsilon_{2}^{\left(0\right)}$, $\varepsilon_{3}^{\left(0\right)}$,…, $\varepsilon_{n}^{\left(0\right)}$) denote the sequence of absolute residual values, where

$$\varepsilon_{k}^{\left(0\right)}=\left|x_{k}^{\left(0\right)}-{\hat{x}}_{k}^{\left(0\right)}\right|,k=2,3,\ldots ,n$$ (11)

Using the same manner of construction as for the original GM(1,1) model for **x**^(0)^, a residual model can be constructed for **ε**^(0)^. The predicted residual, ${\hat{\varepsilon }}_{k}^{\left(0\right)}$, of $\varepsilon_{k}^{\left(0\right)}$ can be derived as follows:

$${\hat{\varepsilon }}_{k}^{\left(0\right)}=(1-e^{a_{\varepsilon }})(\varepsilon_{2}^{\left(0\right)}-\frac{b_{\varepsilon }}{a_{\varepsilon }})e^{-a_{\varepsilon }(k-1)},k=2,3,\ldots ,n$$ (12)

where *a*_*ɛ*_ and *b*_*ɛ*_ are the developing coefficient and the control variable respectively. In the remnant GM(1,1) model with sign estimation, ${\hat{x}}_{k}^{\left(0\right)}$ is modified by adding ${\hat{\varepsilon }}_{k}^{\left(0\right)}$ to, or subtracting ${\hat{\varepsilon }}_{k}^{\left(0\right)}$ from, ${\hat{x}}_{k}^{\left(0\right)}$ [37]:

$${\hat{x}}_{k}^{\left(0\right)}=(1-e^{a})(x_{1}^{\left(0\right)}-\frac{b}{a})e^{-a(k-1)}+s_{k}{\hat{\varepsilon }}_{k}^{\left(0\right)},k=2,3,\ldots ,n$$ (13)

where *s*(*k*) denotes the sign (positive or negative) of ${\hat{\varepsilon }}_{k}^{\left(0\right)}$ with respect to the *k*-th year. Compared to the original remnant GM(1,1) model, the sign of each residual in the improved one is unknown and needs to be estimated.

## Genetic-algorithm-based remnant GM(1,1) model

Two main issues need to be addressed in the traditional remnant GM(1,1) model. First, the determination of both the developing coefficient and the control variable, in the original GM(1,1) model and the residual GM(1,1) model, are completely dependent on the background values. However, as the background values cannot be easily determined in advance by decision makers, it is reasonable to try to find developing coefficients and control variables without using background values. The second issues that needs to be addressed is that *a*_*ɛ*_ and *b*_*ɛ*_ are determined once *a* and *b* in the original GM(1,1) model have been created. However, in addition to sign estimation, to minimize the difference between the predicted and the actual values, it might be worth examining whether simultaneously determining the four crucial parameters (i.e., *a*, *b*, *a*_*ɛ*_, *b*_*ɛ*_) has an effect on the prediction accuracy of the remnant GM(1,1) model.

The objective of our optimization problem is to minimize the mean absolute percentage error (MAPE) of the training patterns:

$$\mathrm{MAPE}=\sum_{k\in TS}^{}\frac{\left|x_{k}^{\left(0\right)}-{\hat{x}}_{k}^{\left(0\right)}\right|}{\left|TS\right|\times x_{k}^{\left(0\right)}}\times 100\%$$ (14)

where *TS* denotes the training or testing data. As the background values are not involved in the formulation of ${\hat{x}}_{k}^{\left(0\right)}$, the computation of MAPE is completely free of the influence of background values. The absolute percentage error (APE), which was used to compare ${\hat{x}}_{k}^{\left(0\right)}$ and $x_{k}^{\left(0\right)}$ with the time series data, was defined as:

$$\mathrm{APE}=\frac{\left|x_{k}^{\left(0\right)}-{\hat{x}}_{k}^{\left(0\right)}\right|}{x_{k}^{\left(0\right)}}\times 100\%$$ (15)

A method based on the GA is developed to automatically determine the developing coefficients (i.e., *a* and *a*_*ɛ*_), the control variables (i.e., *b* and *b*_*ɛ*_), and the sign of the *k*-th year (i.e., *s*_*k*_, *k* = 2, 3, …, *n*) for the improved remnant GM(1,1) model (i.e., GARGM(1,1)). Let *P*_*m*_ denote the population generated in generation *m* (1 ≤ *m* ≤ *n*_*max*_). Chromosome *u* (1 ≤ *u* ≤ *n*_*size*_) produced in *P*_*m*_ is represented as $a_{u}^{m}$ $b_{u}^{m}$ $a_{\varepsilon }{}_{u}^{m}$ $b_{\varepsilon }{}_{u}^{m}$ $s_{u,2}^{m}$ $s_{u,3}^{m}$,…, $s_{u,n}^{m}$ to construct the proposed GARGM(1,1) model, where $a_{u}^{m}$, $b_{u}^{m}$, $a_{\varepsilon }{}_{u}^{m}$, and $b_{\varepsilon }{}_{u}^{m}$ are real-valued genes, and $s_{u,k}^{m}$ is a binary-valued gene for ${\hat{\varepsilon }}_{k}^{\left(0\right)}$. $s_{u,k}^{m}$ has a positive sign when it is one and a negative sign when it is zero.

Let *n*_*size*_ and *n*_*max*_ denote the population size and the maximum number of generations, respectively. Using the MAPE for the training data as the fitness function, having evaluated the fitness value of each chromosome in *P*_*m*_, selection, crossover, and mutation are applied until *n*_*size*_ new chromosomes have been generated for *P*_*m*+1_. The GA can be executed until *n*_*max*_ generations have been generated. The authors of this study performed these genetic operations as described in detail in [38].

### Selection

Using binary tournament selection, two chromosomes from the current population are randomly selected, and the one with the higher fitness is placed in a mating pool. This process is repeated until there are *n*_*size*_ chromosomes in the mating pool. *n*_*size*_ pairs of chromosomes from the pool are then randomly selected for mating. Crossover and mutation operations are applied to a selected parent to reproduce children by altering the chromosomal makeup of the chromosomes of two parents.

### Crossover

For chromosomes *u* ($a_{u}^{m}$ $b_{u}^{m}$ $a_{\varepsilon }{}_{u}^{m}$ $b_{\varepsilon }{}_{u}^{m}$ $s_{u,2}^{m}$ $s_{u,3}^{m}$,…, $s_{u,n}^{m}$) and *v* ($a_{v}^{m}$ $b_{v}^{m}$ $a_{\varepsilon }{}_{v}^{m}$ $b_{\varepsilon }{}_{v}^{m}$ $s_{v,2}^{m}$ $s_{v,3}^{m}$,…, $s_{v,n}^{m}$) (1 ≤ *v* ≤ *n*_*size*_), each pair of real-valued genes has a crossover probability *Pr*_c_. The operations are performed as follows:

$$a_{u}^{m}=h_{1}a_{u}^{m}+(1-h_{1})a_{v}^{m},\;a_{v}^{m}=h_{1}a_{v}^{m}+(1-h_{1})a_{u}^{m};$$

$$b_{u}^{m}=h_{2}b_{u}^{m}+(1-h_{2})b_{v}^{m},\;b_{v}^{m}=h_{2}b_{v}^{m}+(1-h_{2})b_{u}^{m};$$

$$a_{\varepsilon }{}_{u}^{m}=h_{3}a_{\varepsilon }{}_{u}^{m}+(1-h_{3})a_{\varepsilon }{}_{v}^{m},\;a_{\varepsilon }{}_{v}^{m}=h_{3}a_{\varepsilon }{}_{v}^{m}+(1-h_{3})a_{\varepsilon }{}_{u}^{m};$$

$$b_{\varepsilon }{}_{u}^{m}=h_{4}b_{\varepsilon }{}_{u}^{m}+(1-h_{4})b_{\varepsilon }{}_{v}^{m},\;b_{\varepsilon }{}_{v}^{m}=h_{4}b_{\varepsilon }{}_{v}^{m}+(1-h_{4})b_{\varepsilon }{}_{u}^{m};$$

$$s_{u,i}^{m}=h_{k}s_{u,i}^{m}+(1-h_{k})s_{v,i}^{m},\;s_{v,i}^{m}=h_{k}s_{v,i}^{m}+(1-h_{k})s_{u,i}^{m},k=2,\ldots ,n;$$

where *h*_1_, *h*_2_, *h*_3_, *h*_4_, and *h*_*k*_ are all random numbers in the interval [0, 1]. In practice, the one-point crossover operation with *Pr*_c_ is used to exchange partial information between binary-valued substrings (i.e., $s_{u,2}^{m}$ $s_{u,3}^{m}$, …, $s_{u,n}^{m}$ and $s_{v,2}^{m}$ $s_{v,3}^{m}$,…, $s_{v,n}^{m}$) in the selected pair of chromosomes. The crossover point in a substring is chosen randomly. Two new chromosomes are thus generated, and replace their parents in generation *P*_*m*+1_.

### Mutation

Let *Pr*_*m*_ denote the probability that mutation is performed for each real-valued parameter in a new chromosome generated by crossover. To avoid excessive perturbation in the gene pool, a low mutation rate should be used. If mutation occurs for a real-valued gene, it is altered by adding a number randomly selected from a specified interval. For each gene of the newly generated binary chromosomes, the mutation operation with Pr_*m*_ is performed on each bit or gene of the string. Each gene in a string can be thus changed either from zero to one or from one to zero with probability *Pr*_*m*_.

**Fig 1:**
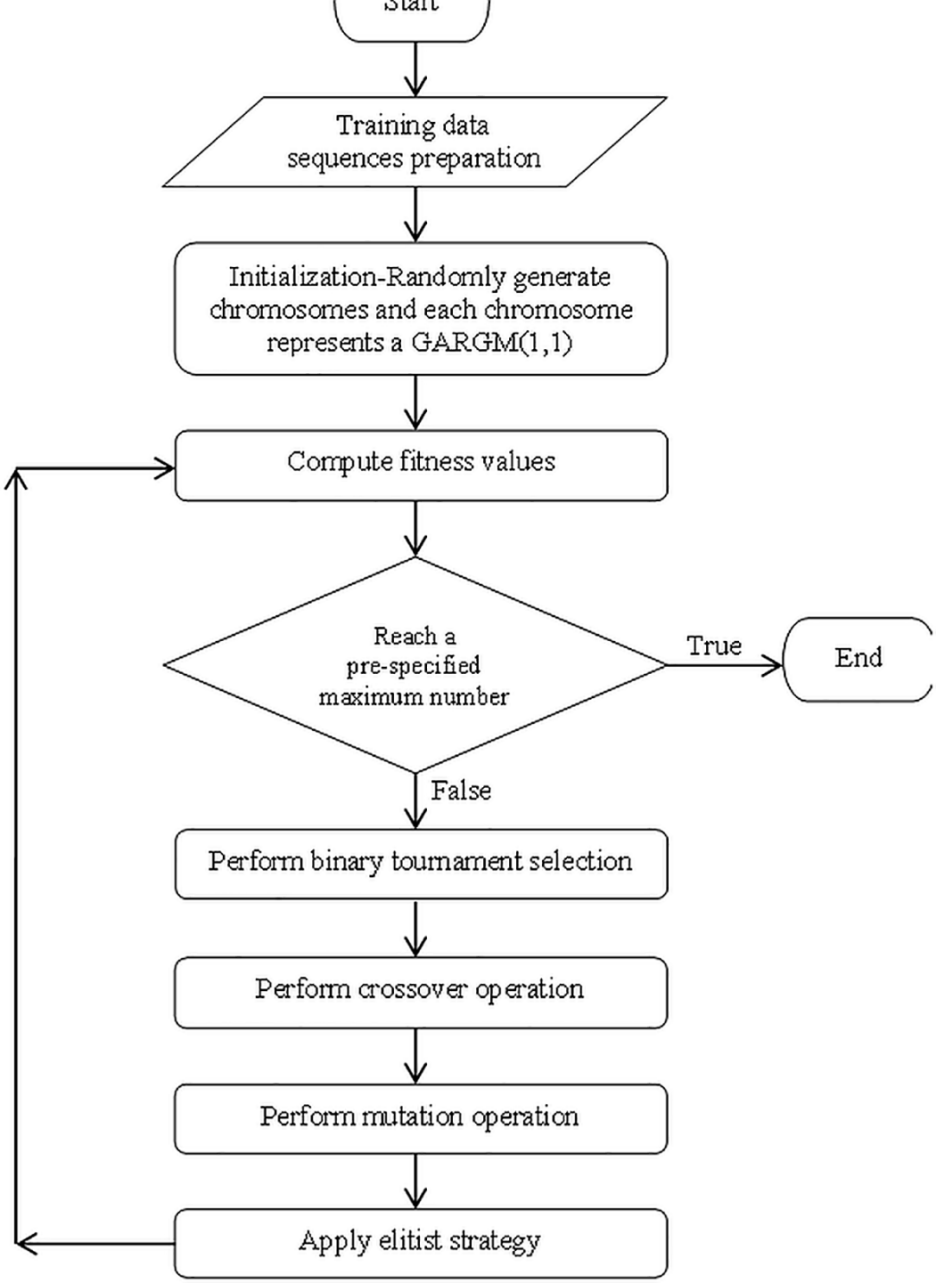
Flowchart of construction of the proposed prediction model.

After crossover and mutation, *n*_*del*_ (0 ≤ *n*_*del*_ ≤ *n*_*size*_) chromosomes in *P*_*m*+1_ are randomly removed from the set of new chromosomes (those formed by genetic operations) to make room for additional copies of the chromosome with a maximum fitness value in *P*_*m*_. Fig 1 shows a flowchart of the construction of the proposed prediction model using the GA.

## Experimental results

Section 4.1 presents the parameter specifications of the GA-based learning algorithm and Section 4.2 reports the performance of different forecasting methods on a real-world case.

### Parameter specifications of GA

A number of factors can influence the performance of the GA, including population size and the probabilities of applying the crossover and mutation operators. As a matter of fact, no optimal GA parameter specifications exist. The principles recommended by Osyczka [35] and Ishibuchi et al. [36] to specify parameters of GA were as follows:

1. The population size commonly should range from 50 to 500 individuals.
2. The stopping condition should be specified according to the available computation time.
3. Only a small number of elite chromosomes were needed.
4. The crossover probability should be set to a large value because it controls the range of exploration in the solution space.
5. The mutation probability should be set to a small value to avoid generating excessive perturbations.

Therefore, the parameters in the experiment were specified as: *n*_*size*_ = 200, *n*_*max*_ = 1000, *n*_*del*_ = 2, *Pr*_*c*_ = 0.9, and *Pr*_*m*_ = 0.01.

This experiment constructed the proposed GARGM(1,1) without any complex mechanisms to tune its parameters.

### Application to total energy demand in China

To examine the forecasting capability of the GARGM(1,1) model, an experiment was conducted to compare its performance with the original GM(1,1), the GPGM(1,1), and the improved grey forecasting model using MLP models (MLPGM(1,1)) on a dataset collected from the China Statistical Yearbook 2008. This dataset made up of historical annual total energy consumption in China was shown in [13]. With its rapid economic development and ongoing industrialization, China has played a vital role with regard to energy production and consumption [39]. Indeed, energy demand forecasting has become an increasingly important issue for China [12].

**Fig 2:**
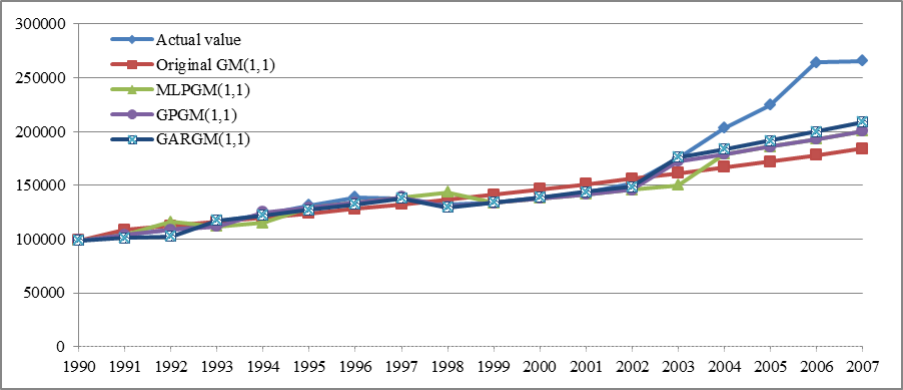
Predicted values and actual values from 1990 to 2007.

Data from 1990 to 2003 were used for model fitting and those from 2004 to 2007 for ex-post testing. The forecasting results reported in [13] for the original GM(1,1), the MLPGM(1,1), and the GPGM(1,1) models are summarized in Table 1 and illustrated in Fig 2. Table 1 shows that the MAPE values of the original GM(1,1), the MLPGM(1,1), the GPGM(1,1), and the GARGM(1,1) models for the training data were 4.13%, 3.61%, 2.59%, and 1.50%, respectively. Similarly, for the testing data, the MAPE values were 26.21%, 20.23%, 20.23%, and 17.51%, respectively. These results indicate that the GARGM(1,1) model outperformed the other forecasting methods on both training and testing data. The results of ex-post testing were relatively poor for every prediction model because the total energy consumption drastically went up in 2004, as shown in Table 1.

**Table 1:** Prediction accuracy obtained by different methods for total energy consumption (unit: 10^4^ tons of SCE).

Year	Actual	Original GM(1,1)		MLPGM(1,1)		GPGM(1,1)		GARGM(1,1)	
		Predicted	APE	Predicted	APE	Predicted	APE	Predicted	APE
1990	98703	98703	0	98703	0	98703	0	98703	0
1991	103783	108706.1	4.74	103783	0	103783	0	101195.7	2.49
1992	109170	112335.5	2.9	116225.8	6.46	108445.2	0.66	102596.3	3.14
1993	115993	116086.1	0.08	111804.1	3.61	111804.1	3.61	117195.8	1.04
1994	122737	119962	2.26	115248.8	6.10	124675.1	1.58	121997.9	0.60
1995	131176	123967.2	5.50	129154.8	1.54	129154.8	1.54	127012.8	3.17
1996	138948	128106.2	7.80	133816.1	3.69	133816.1	3.69	132251.6	4.82
1997	137798	132383.3	3.93	138668.2	0.63	138668.2	0.63	137725.9	0.05
1998	132214	136803.3	3.47	143721	8.7	129885.5	1.76	129692.7	1.91
1999	133831	141370.8	5.63	133756.5	0.06	133756.5	0.06	134280.7	0.34
2000	138553	146090.8	5.44	137709.8	0.61	137709.8	0.61	139002.5	0.32
2001	143199	150968.4	5.43	141743.6	1.02	141743.6	1.02	143858.7	0.46
2002	151797	156008.9	2.77	145855.2	3.91	145855.2	3.91	148849.8	1.94
2003	174990	161217.6	7.87	150041.6	14.26	172393.5	1.48	176275.6	0.73
MAPE			4.13	3.61		2.59		1.50	
2004	203227	166600.2	18.02	178901.5	11.97	178901.5	11.97	183797.7	9.56
2005	224682	172162.6	23.37	185702.4	17.35	185702.4	17.35	191681.9	14.69
2006	264270	177910.7	32.68	192813.8	27.04	192813.8	27.04	199949.4	24.34
2007	265583	183850.7	30.77	200254.3	24.60	200254.3	24.60	208623.1	21.45
MAPE			26.21		20.23		20.23		17.51

Note that Lewis [40] presented the following MAPE criteria for evaluating a forecasting model: MAPE ≤ 10, 10 < MAPE ≤ 20, 20 < MAPE ≤ 50, and MAPE > 50 correspond to high, good, reasonable and weak forecasting models, respectively. Although the original GM(1,1), the GPGM(1,1), the MLPGM(1,1), and the GARGM(1,1) models all have high forecasting capability on the training data, it is noteworthy that the GARGM(1,1) model exhibited its good forecasting capability, but the original GM(1,1), the GPGM(1,1), and the MLPGM(1,1) models recorded merely reasonable forecasting accuracy values on the testing data. It is hence clear that the GARGM(1, 1) model yielded satisfactory performance compared to the other forecasting methods considered.

## Discussion and conclusions

Energy management is crucial for economic prosperity and environmental security [1]. Energy demand forecasting plays an important role in creating energy policy. GM(1,1) is an appropriate approach to predict energy demand because it uses a limited number of samples to construct a prediction model without statistical assumptions. The present study developed the GARGM(1,1) model to forecast energy demand. The proposed model is sufficiently simple to implement as a computer program. Although the parameter specifications are somewhat subjective, the experimental results showed that they are acceptable.

Compared with the MLPGM(1, 1) and the GPGM(1, 1) models, the GARGM(1, 1) model has the advantage of directly determining the developing coefficients and the control variables by the GA without using background values. The required parameters for the RGM are also optimized simultaneously. Experimental results concerning a case of energy demand data from China showed the effectiveness of the proposed forecasting model. In addition to grey prediction models, we examined the prediction performance of two frequently used models, linear regression and the MLP with backpropagation learning. The MLP had an input node, a hidden layer with two neurons, and an output layer with one neuron; it was trained over 10,000 iterations at a learning rate of 0.8. The forecasting results obtained by linear regression and the MLP are summarized in Table 2. It is clear that the prediction accuracy values of linear regression on the training and testing data were 4.20% and 27.76%, respectively [13], whereas those of the MLP on the training and testing data were 3.85% and 18.30%, respectively [20]. Therefore, the proposed GARGM(1,1) outperforms linear regression and the MLP. In case of linear regression, it is reasonable to speculate that the size of the training sample and the statistical assumptions (e.g., homoscedasticity) had a certain impact on the prediction performance of the statistical methods.

**Table 2:** Prediction accuracy obtained by linear regression and MLP.

Year	Actual	Linear regression		MLP		GARGM(1,1)	
		Predicted	APE	Predicted	APE	Predicted	APE
1990	98703	101756.6	3.09	93012.6	5.77	98703	0
1991	103783	106243.4	2.37	107674.6	3.75	101195.7	2.49
1992	109170	110730.2	1.43	116921.0	7.10	102596.3	3.14
1993	115993	115217.0	0.67	122130.4	5.29	117195.8	1.04
1994	122737	119703.8	2.47	125034.6	1.87	121997.9	0.60
1995	131176	124190.6	5.33	126861.3	3.29	127012.8	3.17
1996	138948	128677.5	7.39	128373.0	7.61	132251.6	4.82
1997	137798	133164.3	3.36	130080.1	5.60	137725.9	0.05
1998	132214	137651.1	4.11	132407.0	0.15	129692.7	1.91
1999	133831	142137.9	6.21	135788.9	1.46	134280.7	0.34
2000	138553	146624.7	5.83	140696.7	1.55	139002.5	0.32
2001	143199	151111.5	5.53	147565.7	3.05	143858.7	0.46
2002	151797	155598.3	2.5	156595.8	3.16	148849.8	1.94
2003	174990	160085.1	8.52	167469.6	4.30	176275.6	0.73
MAPE			4.20		3.85		1.50
2004	203227	164572.0	19.02	179212.1	11.82	183797.7	9.56
2005	224682	169058.8	24.76	190465.4	15.23	191681.9	14.69
2006	264270	173545.6	34.33	200083.0	24.29	199949.4	24.34
2007	265583	178032.4	32.97	207546.2	21.85	208623.1	21.45
MAPE			27.76		18.30		17.51

Energy demand forecasting can be regarded as a grey system problem [1, 12] because a few factors, such as income and population, influence energy demand. However, how exactly they influence energy demand is unclear. Therefore, based on the superior forecasting performance of the GARGM(1, 1) model in terms of energy demand, the applicability of the proposed forecasting model to other energy forecasting problems, such as electricity consumption in certain developing countries, should be explored. Moreover, sign estimation for the remnant forecasting model should be explored using other artificial intelligence tools such as the functional-link net [41, 42] and other nonadditive neural networks [43–45] could improve prediction accuracy.

Moreover, it has been known that the GA can be time consuming in searching for optimum solutions. Although forecasting energy demand cannot be treated as a kind of large-scale optimization problem, several improved versions, such as the parallel GA [46–50], may be used to expedite the construction of the model or increase its precision for optimum solutions of the proposed GARGM(1,1) model.
